# Prescribing the dialysis dose and treatment frequency in home haemodialysis

**DOI:** 10.1093/ndt/gfad212

**Published:** 2023-09-26

**Authors:** Francesco Gaetano Casino, Maria Fernanda Slon Roblero, Silvia González-Sanchidrian, Sandra Gallego Dominguez, Ignacio Lorenzo Ferris, Valerie A Luyckx, Vassilios Liakopoulos, Sandip Mitra, Javier Deira Lorenzo, Carlo Basile

**Affiliations:** Dialysis Centre SM2, Policoro, Italy; Nephrology Department, Hospital Universitario de Navarra, Pamplona, Spain; Division of Nephrology, San Pedro de Alcantara Hospital, Cáceres, Spain; Division of Nephrology, San Pedro de Alcantara Hospital, Cáceres, Spain; Nephrology Department, Hospital Universitario de Navarra, Pamplona, Spain; Renal Division, Brigham and Women's Hospital, Harvard Medical School, Boston, MA, USA; Department of Paediatrics and Child Health, University of Cape Town, South Africa; Department of Public and Global Health, Epidemiology, Biostatistics and Prevention Institute, University of Zurich, Zurich, Switzerland; Second Department of Nephrology, AHEPA University Hospital, Medical School, Aristotle University of Thessaloniki, Thessaloniki, Greece; Manchester Academy of Health Sciences Centre (MAHSC), Manchester University Hospitals and University of Manchester, Manchester, UK; Division of Nephrology, San Pedro de Alcantara Hospital, Cáceres, Spain; Associazione Nefrologica Gabriella Sebastio, Martina Franca, Italy

**Keywords:** dialysis rhythms, eKt/V, home haemodialysis, stdKt/V, urea kinetic model

## Abstract

**Background:**

There is growing interest in home haemodialysis (HHD) performed with low-flow dialysate devices and variable treatment schedules. The target standard Kt/V (stdKt/V) should be 2.3 volumes/week, according to KDOQI guidelines (2015). The current formula for stdKt/V does not help prescribe the dialysis dose (eKt/V) and treatment frequency (TF). The aim of this study was to obtain a formula for stdKt/V that is able to define the minimum required values of eKt/V and TF to achieve the targeted stdKtV.

**Methods:**

Thirty-eight prevalent patients on HHD were enrolled. A total of 231 clinical datasets were available for urea modelling using the Solute-Solver software (SS), recommended by KDOQI guidelines. A new formula (stdKt/V = a + b × Kru + c × eKt/V) was obtained from multivariable regression analysis of stdKt/V vs eKt/V and residual kidney urea clearance (Kru). The values of coefficients a, b and c depend on the treatment schedules and the day of the week of blood sampling for the kinetic study (labdayofwk) and then vary for each of their foreseen 62 combinations. For practical purposes, we used only seven combinations, assuming Monday as a labdayofwk for each of the most common schedules of the 7 days of the week.

**Results:**

The stdKt/V values obtained with SS were compared with the paired ones obtained with the formula. The mean ± standard deviation stdKt/V values obtained with SS and the formula were 3.043 ± 0.530 and 2.990 ± 0.553, respectively, with 95% confidence interval +0.15 to –0.26. A ‘prescription graph’ was built using the formula to draw lines expressing the relationship between Kru and required eKt/V for each TF. Using this graph, TF could have been reduced from the delivered 5.8 ± 0.8 to 4.8 ± 0.8 weekly sessions.

**Conclusions:**

The new formula for stdKtV is reliable and can support clinicians to prescribe the dialysis dose and TF in patients undergoing HHD.

## Graphical Abstract

**Graphical Abstract:**
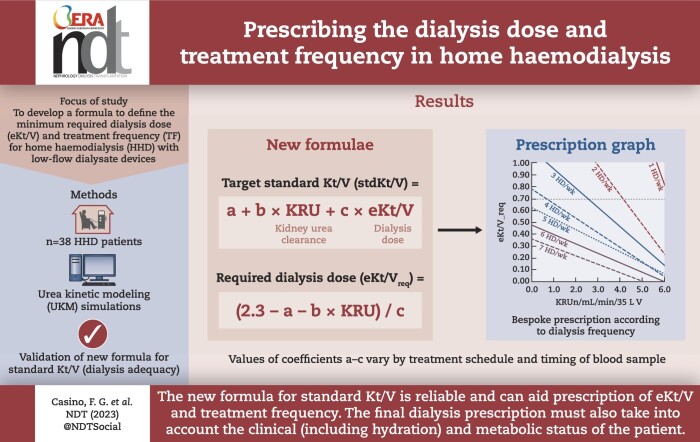


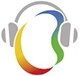
 Watch the video of this contribution at https://academic.oup.com/ndt/pages/author_videos

KEY LEARNING POINTS **What was known:**

- There is increasing interest in home haemodialysis (HHD) performed with low-flow dialysate devices and variable treatment schedules.
- The 2015 KDOQI guidelines suggest a single target of standard Kt/V (stdKt/V) of 2.3 volumes/week, with a minimum of 2.1, but do not provide a guide for computing the dialysis dose (eKt/V) and treatment frequency to be prescribed.
- The dialysis prescription based on urea kinetic modelling, which requires the measurement of residual kidney urea clearance (Kru), could help prescribe the dialysis dose and treatment frequency in patients undergoing HHD.

**This study adds:**

- A new formula for stdKt/V was obtained: stdKt/V = a + b × Kru + c × eKt/V. It estimates stdKt/V from Kru, urea distribution volume (V), eKt/V and a set of three coefficient values specific for the treatment frequency.
- The stdKtV values estimated with the new formula are tightly correlated with the paired ones obtained using the Solute-Solver software, recommended by the 2015 KDOQI guidelines, with a squared Pearson coefficient (R^2^) > 0.96 and with 95% confidence interval ranging from +0.15 to –0.26 volumes/week.
- The new formula can be used to directly provide the required eKt/V to achieve the targeted stdKt/V, as a function of Kru and treatment frequency. On this basis, a ‘prescription graph’ was built, that allows to identify the minimum dialysis dose and treatment frequency necessary to achieve the target stdKt/V.

**Potential impact:**

- Our formula for stdKtV can help prescribe the dialysis dose and treatment frequency in patients undergoing HHD performed with low-flow dialysate devices. The formula can be extended to other clinical settings because it is based on the mathematical relationship existing between stdKt/V and eKt/V. In other words, it can be used regardless of the actual blood or dialysate flow rates, at least in the usual clinical ranges.
- Our method aims at estimating the minimum dialysis requirements based on depurative purposes. The final dialysate prescription must also take into account the hydration, clinical and metabolic status of the patient.

## INTRODUCTION

Recently, there has been growing interest in more frequent haemodialysis (HD) rhythms, especially in the setting of home haemodialysis (HHD). An important factor contributing to the diffusion of HHD was the introduction of simple and portable devices specially designed for this setting [1–5]. Typically, 5 or 6 weekly sessions are delivered with these portable monitors, using a low dialysate flow rate for 2–3 h per session [1–5]. This technique allows the dialysis treatment to be easily adapted to changing patient needs, which implies a wide range of schedules and treatment times. In the absence of specific evidence, the prescription of dialysis dose (eKt/V) in patients undergoing HHD is currently based on the general criteria proposed by the KDOQI 2015 guidelines, which suggest a single target for the total (renal + dialysis) weekly clearance, as expressed by a standard Kt/V (stdKt/V) value equal to 2.3 volumes/week (v/wk), with a minimum delivered dose not less than 2.1 v/wk, for all HD schedules [6]. In particular, the KDOQI guidelines recommend measuring stdKt/V with the double pool urea kinetic model (UKM), using either the Solute-Solver software (SS) (version 2.14, currently available at www.ureakinetics.org) [7] or the formula proposed by the Frequent Hemodialysis Network (FHN), which takes into account ultrafiltration and kidney urea clearance (Kru) evaluated at 100% [8]. The FHN formula is certainly easier to use than SS for evaluating the amount of dialysis delivered over a 1-week period; however, it is somewhat complex and cannot be easily solved to predict the dialysis dose (eKt/V) required to achieve the target stdKt/V value of 2.3 v/wk (eKt/V_req).

Very recently, we published a study that aimed to validating formulas calculating normalized protein catabolic rate (PCRn) in patients undergoing HHD performed with low-flow dialysate devices [9]. As a further development of this kinetic modelling study, we explored the possibility of establishing a new formula for estimating stdKt/V that can be easily solved to provide the required dialysis dose (eKt/V_req) to achieve the stdKt/V target of 2.3 in patients undergoing HHD.

## MATERIALS AND METHODS

### Subjects

Thirty-eight prevalent patients on maintenance dialysis, being treated with HHD at the Division of Nephrology of University Hospital of Navarra, Pamplona, Spain (24 patients) and at the Division of Nephrology of San Pedro de Alcántara Hospital, Cáceres, Spain (14 patients), were enrolled into the present study. The main baseline clinical and treatment data of the 38 patients studied are reported in Table 1. Thirty-three of them had a tunnelled central venous catheter. All patients gave verbal and written informed consent for the choice of HHD as modality of kidney replacement therapy and for participation in the present study. The latter was approved for the two participating centres by the Cáceres Ethics Committee. Inclusion criteria were: (i) already being on HHD; and (ii) the availability of at least one complete dataset for UKM analysis with SS [7]. Most patients were on frequent dialysis regimens; various combinations of schedules and treatment times were used to obtain a stdKt/V higher than 2.1 [9]. The HHD sessions were performed with Physidia S3 monitors (PALEX^®^, Spain) and NxStage^®^ SystemOne monitors (Fresenius Medical Care, Spain).

**Table 1:** Baseline clinical data of the 38 patients enrolled into the study (at their first available urea kinetic study on HHD).

ID	Age (years)	Body weight (kg)	Body mass index (kg/m^2^)	Months on dialysis	Urinary output (L/day)	Treatment time (min)	HD sessions per week
1	36	73	25.9	14	0	150	5
2	58	77.5	27.1	7	0.8	150	5
3	50	67	24	27	0	150	5
4	63	60.5	23.1	35	0	145	5
5	69	59.5	23.5	5	0.85	150	5
6	58	58	25.7	29	0	145	5
7	46	59	23.9	67	0	175	4
8	56	94	29	3	2.4	145	3
9	56	109.6	35.78	5	2.7	145	3
10	62	82.5	24.1	11	0.9	150	4
11	50	105	30.7	40	0	180	5
12	33	62.7	23.4	46	0	150	5
13	31	41.5	17.3	8	0	150	4
14	39	64	21.6	168	0	150	5
15	46	130.6	38.6	52	0	150	7
16	64	76.6	31.1	264	0	150	6
17	60	59.9	25.6	144	0	150	6
18	38	109.7	36.2	55	0	150	7
19	58	84.1	27.1	62	0	120	6
20	71	68.8	31.8	108	0	150	5
21	57	80	25.3	50	0	150	6
22	32	52.3	16.5	20	0	150	6
23	34	84.4	31.4	156	0	150	6
24	42	49.3	21.9	49	0	150	5
25	69	77.4	28.8	60	0	150	7
26	59	58.9	19.9	432	0	150	6
27	28	52.1	20.4	372	0	150	6
28	74	66	23.9	34	0	150	6
29	66	71.5	23.6	96	0	150	6
30	56	74.4	28.4	29	0	120	6
31	42	65.4	24.3	192	0	150	5
32	66	58.1	25.8	28	0	150	5
33	43	49.9	22.1	48	0	150	5
34	44	49.9	18.4	192	0	150	5
35	61	90.3	25.8	468	0	180	6
36	46	57.8	17.8	72	0	150	7
37	22	65.2	18.1	7	0	150	6
38	72	74	22.6	90	0.6	150	5

### Methods

A total of 231 clinical datasets suitable for UKM analysis and associated with available bimonthly monitoring sessions were retrieved from local electronic clinical databases. It must be underlined that the basic methodological concepts developed in our recent paper constitute the background of the present study [9].

#### Dialysis simulation plan

As detailed elsewhere [9], we prepared a dialysis simulation plan. To illustrate the structure of our simulation plan, carried out with the version 1.19 of the ‘What-if’ module of SS [10], it is useful to specify that the input dataset of this software requires, among other things, the indication of the treatment schedule, i.e. the sequence of days in which the dialysis run is scheduled, numbered from 1 (Monday) to 7 (Sunday), and the day on which blood samples were drawn for the kinetic study (labdayofwk). For example, the sequence 135 indicates a thrice a week (3 HD/week) schedule with HD sessions performed on Monday, Wednesday and Friday. However, a 3 HD/week timetable could also be, for example, 146, i.e. Monday, Thursday and Saturday. In addition, the day on which the blood samples were drawn (labdayofwk) must also be indicated: it must be one of the dialysis days, so that the software can calculate the right sequence of short and long interdialytic intervals. For example, labdayofwk = 1 indicates that the blood sample was drawn on Monday. Being aware that HHD patients can have a much wider choice of schedules than in-centre HD patients, we have foreseen a wide range of treatment sequences. Since the day on which the blood samples are drawn can also vary, the number of possible combinations of schedule and labdayofwk, which for convenience we call ‘simulation units’, can be very high. In order to set an appropriate simulation plan we considered 62 simulation units to include almost all possible realistic combinations of schedules and labdayofwk. For each basic simulation unit, constant values were set for the following parameters: urea distribution volume (V = 35 L), blood flow rate (Qb = 350 mL/min), dialysate flow rate (Qd = 180 mL/min) and weekly ultrafiltration (WeeklyUF = 10 L). For greater realism, WeeklyUF was set to l L for the once a week schedule. We varied the following input data for each simulation unit, one at a time: residual kidney urea clearance (Kru: four values: from 0 to 6.0 mL/min; step: 2 mL/min); dialyser urea clearance (Kd, five values: from 100 to 200 mL/min; step: 25 mL/min); generation rate (G, six values: from 2.78 to 9.76 mg/min, to get a PCRn from 0.6 to 1.6; step: 0.2 g/kg/day); session length [treatment duration (Td), four values: 120, 150, 180, 200 min]. As a result, there were 4 × 5 × 6 × 4 = 480 different input datasets for each of the 62 simulation units.

#### Statistics

Means ± standard deviation (SD), Bland–Altman plot and simple linear regressions were performed with Excel^®^; Student's *t*-test for paired data and multiple linear regressions were performed with the Jamovi statistical software [11, 12].

## RESULTS

**Figure 1:**
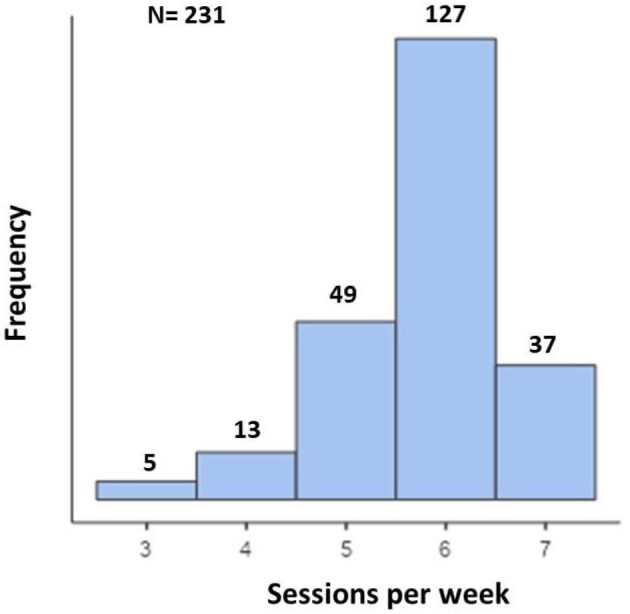
Histogram showing the frequency of dialysis sessions a week.

Table 2 shows the means ± SD of the most relevant input and output data of SS concerning the available 231 clinical datasets. The most common treatment frequency was 6 sessions a week (55%), followed by 7 sessions a week (16%) and 5 sessions a week (21%) (Fig. 1). A mean stdKt/V value of 3.040 v/wk was observed, meaning that in many cases a much higher dose of dialysis than required was provided. Indeed, stdKt/V was >2.3 in 213 of the 231 sessions examined (92%) (Table 2).

**Table 2:** Relevant input and output data of SS (*N* = 231) as well as some relevant biochemical data.

	Qb mL/min	Qd mL/min	Td min	Post-HD body weight kg	UF L/session	eKt/V per session	Urea distribution volume (L)	stdKt/V v/wk	Pre-HD P	Post-HD P	Pre-HD β_2_	Post-HD β_2_
Mean	343	185	152	73.6	1.0	0.677	33.0	3.04	5.11	2.67	27.2	14.8
SD	26.2	18	13.1	18.3	0.63	0.163	9.03	0.53	1.05	0.94	5.81	3.11

Urine output was present in only 20 datasets; Kru was 4.0 ± 3 0.0 mL/min.

UF: ultrafiltration; P: serum phosphate values (mg/dL); β_2_: serum β_2_-microglobulin values (μg/mL).

By computer simulation of weekly dialysis cycles with changing values of Kru and eKt/V, we first computed a series of associated stdKt/V values and then established the regression of stdKt/V vs the paired Kru and eKt/V values, for each combination of schedule and labdayofwk. As a result, we got the following general equation:


(1) \begin{equation*}
{\mathrm{stdKt}}/{\mathrm{V}} = {\mathrm{a}} + {\mathrm{b}} \times {\mathrm{Kru}} + {\mathrm{c}} \times {\mathrm{eKt}}/{\mathrm{V}}
\end{equation*}

where the values of coefficients a, b and c depend on the treatment schedule and labdayofwk and then vary for each of the 62 foreseen combinations of schedules and labdayofwk.

The coefficient b in Equation 1 refers to a patient with a typical V of 35 l. Therefore, Kru value in this case is mL/min for V = 35 l. To use Equation 1 also in patients with V ≠ 35 l, normalized Kru (KRUn = Kru/V × 35) can be used [13].

Thus, stdKt/V becomes:


(1) \begin{equation*}{\mathrm{stdKt}}/{\mathrm{V}} = {\mathrm{a}} + {\mathrm{b}} \times {\mathrm{KRUn}} + {\mathrm{c}} \times {\mathrm{eKt}}/{\mathrm{V}}\end{equation*}

Of note, it must be stressed that Kru and KRUn values to be used with UKM-derived formulas should always be expressed in term of blood water, as are the urea concentrations used by UKM.

In order to simplify the issue, we used only seven combinations, one for each day of the week, all with labdayofwk = 1, i.e. on Monday (Table 3). The complete list of the 62 foreseen combinations is shown in the Supplementary data, Table S1). When using the short list of seven combinations, values of stdKt/V were 2.990 ± 0.553, very close to the ones obtained with the 62 combinations: 3.043 ± 0.530 (mean difference –0.053 ± 0.023).

**Table 3:** Values of the coefficients a, b and c of Equation 1 for schedules ranging from 1 to 7 sessions a week, assuming a fixed labdayofwk = 1, i.e. on Monday.

Number of weekly treatments	A	b	c
1	0.126	0.288	0.543
2	0.234	0.288	1.201
3	0.324	0.288	1.781
4	0.471	0.288	2.335
5	0.358	0.289	3.098
6	0.565	0.289	3.604
7	0.827	0.289	4.089

**Figure 2:**
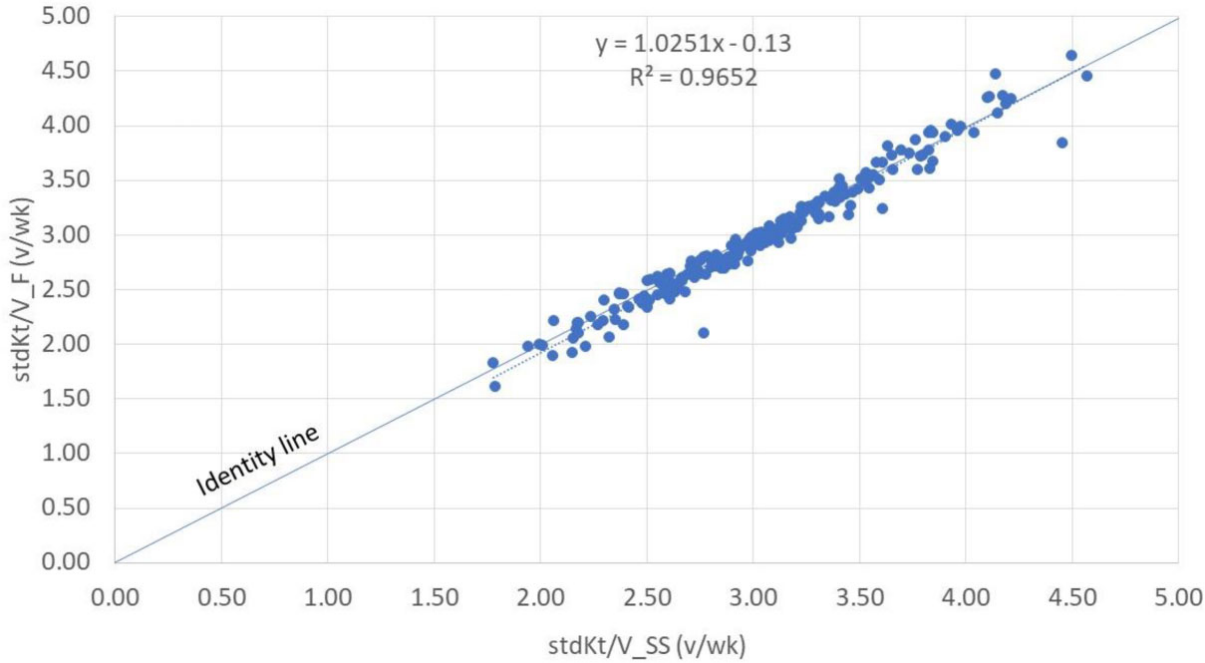
Regression line of stdKt/V values estimated with the formula (F) and the paired ones computed with SS, using the simplified set of coefficients.

As shown in Fig. 2, there was an excellent correlation between the paired stdKt/V values, with the regression line virtually coincident with the identity line (R^2^ > 0.96). For completeness, we report the correlation between the stdKt/V values obtained using all 62 combinations and the paired ones calculated with the reference SS (Supplementary data, Fig. S1). Also in this case, there was an excellent correlation between the paired stdKt/V values, with the regression line virtually coincident with the identity line (R^2^ > 0.97).

**Figure 3:**
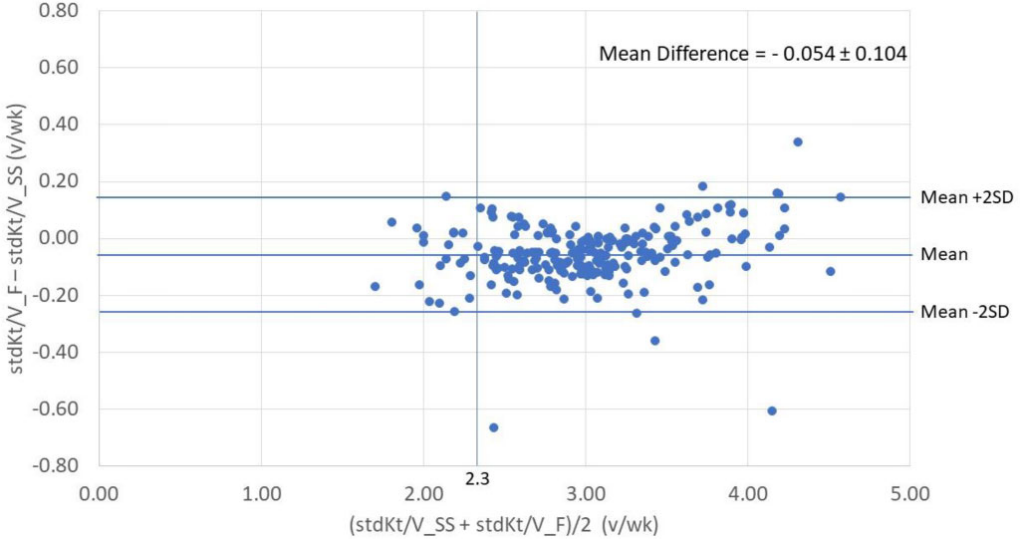
Agreement plot (Bland–Altman) of stdKt/V values estimated with the formula (F) and the paired ones computed with SS, using the simplified set of coefficients.

To validate Equation 1, the available 231 clinical datasets were used to evaluate the agreement between the stdKt/V values provided by Equation 1 (stdKt/V_F) and the paired ones calculated with the reference SS (stdKt/V_SS). The agreement plots of stdKt/V estimated with the formula and the paired ones computed with SS were quite similar: the mean difference was –0.054 ± 0.104 (95% confidence interval +0.15 to –0.26), when using the list of the 7 combinations (Fig. 3) and –0.003 ± 0.087 v/wk (95% confidence interval +0.17 to –0.18) when using the list of the 62 combinations (Supplementary data, Fig. S2). The vertical line in correspondence of stdKt/V = 2.3 v/wk shows that the vast majority of patients received a higher dialysis dose than the one required to achieve the stdKt/V target of 2.3 v/wk (Fig. 3 and Supplementary data, Fig. S2).

By solving Equation 1 for eKt/V, one gets:


(2) \begin{equation*}{\mathrm{eKt}}/{\mathrm{V}} = \left( {{\mathrm{stdKt}}/{\mathrm{V}}-{\mathrm{a}}-{\mathrm{b}} \times {\mathrm{KRUn}}} \right)/{\mathrm{c}}\end{equation*}

By replacing the actual stdKt/V value with the target value of 2.3, the eKt/V provided by Equation 2 becomes eKt/V_req, that is the dialysis dose required to achieve the goal. Moreover, since the above equations have a general meaning, to apply them in real patients, one must use the specific coefficient values for the appropriate schedule-labdayofwk combination (Table 3).

Thus, the equation for eKt/V_req is:


(3) \begin{equation*}{\mathrm{eKt}}/{\mathrm{V}}\_{\mathrm{req}}\ = \left( {2.3-{\mathrm{a}}-{\mathrm{b}} \times {\mathrm{KRUn}}} \right)/{\mathrm{c}}\end{equation*}

As a practical example, for a patient on 5 sessions a week with a schedule 12345 and blood sampling on Monday, the values of the coefficients a, b and c shown in Table 3 are 0.358, 0.289 and 3.098, respectively, and if he/she has Kru = 2.0 mL/min, V = 30 l and eKt/V = 0.6, one can get:


\begin{eqnarray*}
{\mathrm{stdKt}}/{\mathrm{V}} = 0.358 + 0.289 \times 2/30 \times 35 + 3.098 \times 0.6 = 2.89\,\,{\mathrm{v}}/{\mathrm{wk}}
\end{eqnarray*}

**Figure 4:**
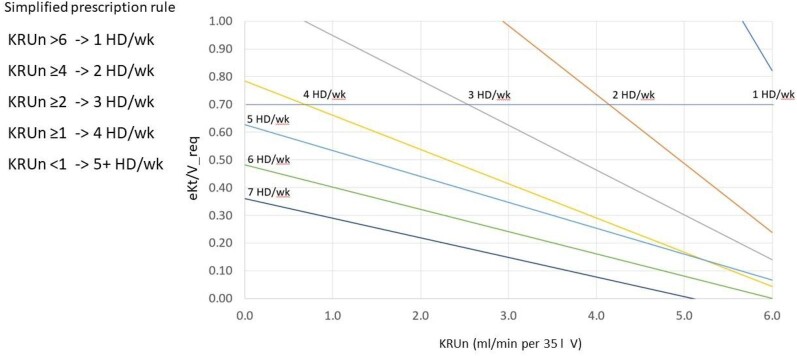
The prescription graph shows that treatment frequency depends essentially on KRUn.

Figure 4 shows the plot of eKt/V_req as a function of KRUn and treatment frequency. The intersection of the horizontal line with ordinate 0.7, corresponding to the mean value of eKt/V observed in the study, indicates the minimum KRUn (cut-off) required to obtain stdKt/V of 2.3 giving an eKt/V of 0.7. Simplifying, the following rule of thumb can be established: 1 HD/week is possible with KRUn >6 mL/min; 2 HD/week with KRUn ≥4 mL/min; 3 HD/week with KRUn ≥2 mL/min; 4 HD/week with KRUn ≥1 mL/min; for values of KRUn <1 mL/min for V = 35 l, 5 HD/week are sufficient to achieve the target stdKt/V of 2.3 with an eKt/V of 0.6; finally, for 6 and 7 HD/week an eKt/V of 0.5 and 0.4, respectively, are more than enough.

Combining the above rule with Equation 3, we recalculated the minimum of treatment frequency and associated eKt/V_req for each of the available 231 clinical datasets. Table 4 compares the number of sessions delivered for each schedule with the number of sessions for each schedule to be prescribed according to our method (see the Explanatory Note to Table 4 and Fig. S3 in the Supplementary data).

**Table 4:** Comparison of the number of sessions delivered for each schedule with the number of sessions for each schedule to be prescribed according to our method.

Sessions per week	1	2	3	4	5	6	7	Mean ± SD
Delivered	0	0	5	13	49	127	37	5.8 ± 0.86
To be prescribed	7	1	5	6	**212** ^a^	0	0	4.8 ± 0.77^b^

a See the Explanatory Note to Table 4 and Fig. S3 in the Supplementary data.

b Student's *t*-test for paired data (*P* < .001).

## DISCUSSION

The ultimate goal for patients on dialysis is prolongation of life with the best achievable quality of life. Here we want to underline that the goal of dialysis adequacy is only a part of the adequacy of care of the patient on dialysis. Dialysis-dependent patients require the solution of several clinical and metabolic problems, which are independent of or only partially dependent on the dialysis adequacy *per se*. Many of these problems develop long time before the start of dialysis.

The quest for a reliable dialysis adequacy index/criteria has been a constant through the decades of dialysis. Recent publications reflect the shift in nephrologists’ understanding of dialysis adequacy and reinforce the idea that a new approach needs to be considered [14], moving away from a ‘one-size-fits-all’ approach to dialysis to more personalized care that incorporates patient goals and preferences while maintaining best practices for quality and safety [15]. Nearly 60 years after the first clinical use of HD there is still no consensus on how to prescribe some important aspects of HD such as treatment duration and frequency of dialysis sessions [14]. This is in part due to conventional HD practice being largely limited to a rigid 3 HD/week schedule. Recently, there has been a growing interest in more frequent dialysis schedules and personalized routines, especially in the setting of HHD. Technological advances have led to a wider use of HHD performed with low-flow dialysate devices, thus increasing its acceptance amongst patients due to miniaturization and simplification of devices and water treatment apparatus, but nevertheless raising the question of how these systems can be deployed to achieve an adequate dialysis dose adapted to the specific characteristics of the limited flow dialysate treatments. The ability to prescribe flexible but adequate dialysis schedules during the week allows personalization of the treatment and offers a huge incentive for greater uptake of HHD by patients.

Being believers or not of the crucial role plaid by UKM in the dialysis prescription, in the absence of specific evidence, the prescription of dialysis dose (eKt/V) in patients undergoing HHD is currently based on the general criteria proposed by the KDOQI 2015 guidelines, which suggest a single target for the total (renal + dialysis) weekly clearance, as expressed by a stdKt/V value equal to 2.3 v/wk, with a minimum delivered dose not less than 2.1 v/wk for all HD schedules [6]. These guidelines [6] and the more recent Renal Association (UK) clinical practice guideline on HD [16] recommend monitoring of dialysis dose on a monthly basis using eKt/V as the most clinically valid small solute measure of dialysis dose [evidence level 1B]. Thus, these measurements should be performed in both centre-based and HHD patients. The aim of our work was to facilitate this monitoring by the adoption of the new formula.

What about the generalizability of the new formula? While acknowledging that it was validated in a very particular data setting (a relatively high blood flow rate, a low dialysate flow rate and a short duration of the session), we can state without doubt that it can be extended to other clinical settings. In fact, the formula is based on the mathematical relationship that exists between stdKt/V and eKt/V. Thus, what matters is the value of eKt/V, not how it is obtained. In other words, it can be used regardless of the actual blood or dialysate flow rates, at least in the usual clinical ranges.

The prescription graph demonstrates the key role of Kru in setting the treatment frequency: as Kru declines, the treatment frequency should increase to achieve the target stdKt/V of 2.3 v/wk. Thus, great attention must be paid when dialysis prescription largely depends on a high residual kidney function. The consequent warning is that close monitoring of urine output and Kru are mandatory.

Our study shows that the stdKt/V achieved was >2.3 v/wk in 213 of the 231 sessions examined (92%). On practical grounds, using our prescription graph, we realized that treatment frequency could have been reduced from the delivered 5.8 ± 0.8 to 4.8 ± 0.8 weekly sessions. As shown in Table 4, at least in principle, our prescription method could reduce the schedules with 6 or 7 session a week to a schedule with only 5 sessions a week. This is a direct consequence of the ‘simplified prescribing rule’ given in Fig. 4, whereby an anuric patient could easily achieve the target stdKt/V of 2.3 v/wk by being prescribed an eKt/V as low as 0.6 per 5 sessions a week. We would stress that the above prescription approach is based only on considerations of urea kinetics, but in clinical practice there are many possible reasons to increase the treatment frequency, such as a marked increase in interdialytic body weight, not controllable by increasing the dose of diuretics, and symptoms or signs of uraemia, such as nausea or malnutrition, refractory to medical therapy.

On the other hand, it must be underlined that the target stdKt/V of 2.3 v/wk probably overestimates the required dialysis dose. In fact, a stdKt/V of 2.3 should correspond to a relatively high level of Kru with KRUn = 2.3 × 35 000 mL/10 080 min = 8 mL/min, which on average corresponds to a kidney creatinine clearance of 16 mL/min and a glomerular filtration rate (GFR) of 12 mL/min/1.73 m^2^. It is worth noting that Canadian guidelines suggest starting dialysis with a GFR of approximately 6 mL/min/1.73 m^2^ [17]. In this regard, it is important to underline once more that the prescription of HD should not be based on a single parameter, such as stdKt/V, but also on other determinants such as volume control, biochemical parameters, nutritional status, cardiovascular function and symptoms.

In conclusion, our formula for stdKtV is reliable and can be used in clinical practice to prescribe the dialysis dose and treatment frequency in patients undergoing HHD with novel HD technology performed with low-flow dialysate devices. The availability of this simple method could be useful to adjust the frequency of treatment to personalize schedules at home in the interest of both the patient, prescribers and the healthcare system. This will allow safer prescribing of variable schedules in the home setting, and the use of the advanced dialysis technology, both as necessary drivers to promote a greater uptake of HHD. However, it must be recognized that our method aims at estimating the minimum dialysis requirements based on depurative purposes, whilst the clinical judgment of the attending nephrologist remains the final determinant of the dialysis prescription. The latter must take into account and match evidence with patient preferences.

## Supplementary Material

gfad212_Supplemental_File

## Data Availability

The data underlying this article will be shared on reasonable request to the corresponding author.
