# Supplementary material for: Prescribing the dialysis dose and treatment frequency in home haemodialysis
Source: Nephrol Dial Transplant. 2023 Sep 26;39(3):445–52. doi: 10.1093/ndt/gfad212 (PMC10939408; doi:10.1093/ndt/gfad212)

**Prescribing the dialysis dose and treatment frequency in home haemodialysis**

^1^Francesco Gaetano Casino, ^2^Maria Fernanda Slon Roblero, ^3^Silvia González-Sanchidrian, ^3^Sandra Gallego Dominguez, ^2^Ignacio Lorenzo Ferris, **^4^Valerie A. Luyckx, ^5^Vassilios Liakopoulos,** ^6^Sandip Mitra, ^3^Javier Deira Lorenzo and ^7^Carlo Basile on behalf of the EuDial Working Group of the European Renal Association (ERA)

Corresponding author:

Carlo Basile, MD

Via Battisti 192

74121 Taranto - ITALY

Tel + 39-099-4773688

Mobile + 39-3290628486

E-mail: [basile.miulli@libero.it](mailto:basile.miulli@libero.it)

**SUPPLEMENTARY MATERIAL**

Table 1S. Values of the coefficients of Equation 1 for the 62 combinations of treatment schedules and labdayofwk considered in the study.

| **Schedule**  **& labday** | **a** | b | **c** | **Schedule**  **& labday** | **a** | **b** | **c** |
| --- | --- | --- | --- | --- | --- | --- | --- |
| 1.1 | 0.126 | 0.288 | 0.543 | 12345.1 | 0.358 | 0.289 | 3.098 |
| 14.1 | 0.234 | 0.288 | 1.201 | 12345.2 | 0.666 | 0.289 | 2.892 |
| 14.4 | 0.296 | 0.288 | 1.159 | 12345.3 | 0.672 | 0.289 | 2.878 |
| 15.1 | 0.296 | 0.288 | 1.159 | 12345.4 | 0.674 | 0.289 | 2.872 |
| 15.5 | 0.234 | 0.288 | 1.201 | 12345.5 | 0.674 | 0.289 | 2.869 |
| 25.2 | 0.234 | 0.288 | 1.201 | 12356.1 | 0.521 | 0.289 | 2.965 |
| 25.5 | 0.296 | 0.288 | 1.159 | 12356.2 | 0.67 | 0.289 | 2.861 |
| 26.2 | 0.296 | 0.288 | 1.159 | 12356.3 | 0.674 | 0.289 | 2.851 |
| 26.6 | 0.234 | 0.288 | 1.201 | 12356.5 | 0.521 | 0.289 | 2.964 |
| 135.1 | 0.324 | 0.288 | 1.781 | 12356.6 | 0.67 | 0.289 | 2.86 |
| 135.3 | 0.412 | 0.289 | 1.723 | 12456.1 | 0.521 | 0.289 | 2.964 |
| 135.5 | 0.412 | 0.289 | 1.721 | 12456.2 | 0.67 | 0.289 | 2.86 |
| 246.2 | 0.324 | 0.288 | 1.781 | 12456.4 | 0.521 | 0.289 | 2.965 |
| 246.4 | 0.412 | 0.289 | 1.723 | 12456.5 | 0.67 | 0.289 | 2.861 |
| 246.6 | 0.412 | 0.289 | 1.721 | 12456.6 | 0.674 | 0.289 | 2.851 |
| 146.1 | 0.412 | 0.289 | 1.721 | 13456.1 | 0.519 | 0.289 | 2.967 |
| 146.4 | 0.324 | 0.288 | 1.781 | 13456.3 | 0.518 | 0.289 | 2.971 |
| 146.6 | 0.412 | 0.289 | 1.723 | 13456.4 | 0.669 | 0.289 | 2.868 |
| 1245.1 | 0.344 | 0.289 | 2.432 | 13456.5 | 0.673 | 0.289 | 2.856 |
| 1245.2 | 0.584 | 0.288 | 2.272 | 13456.6 | 0.674 | 0.289 | 2.853 |
| 1245.4 | 0.471 | 0.289 | 2.344 | 123456.1 | 0.565 | 0.289 | 3.604 |
| 1245.5 | 0.586 | 0.289 | 2.265 | 123456.2 | 0.748 | 0.289 | 3.483 |
| 1246.1 | 0.471 | 0.288 | 2.335 | 123456.3 | 0.753 | 0.289 | 3.471 |
| 1246.2 | 0.587 | 0.288 | 2.252 | 123456.4 | 0.565 | 0.289 | 3.604 |
| 1246.4 | 0.472 | 0.288 | 2.332 | 123456.5 | 0.754 | 0.289 | 3.467 |
| 1246.6 | 0.471 | 0.288 | 2.334 | 123456.6 | 0.753 | 0.289 | 3.466 |
| 1356.1 | 0.472 | 0.288 | 2.332 | 1234567.1 | 0.827 | 0.289 | 4.089 |
| 1356.3 | 0.471 | 0.288 | 2.334 | 1234567.2 | 0.827 | 0.289 | 4.089 |
| 1356.5 | 0.471 | 0.288 | 2.335 | 1234567.3 | 0.827 | 0.289 | 4.089 |
| 1356.6 | 0.587 | 0.288 | 2.252 | 1234567.4 | 0.827 | 0.289 | 4.089 |
|  |  |  |  | 1234567.5 | 0.827 | 0.289 | 4.089 |
|  |  |  |  | 1234567.6 | 0.827 | 0.289 | 4.089 |

Figure 1S. Regression line of stdKt/V values estimated with the formula (F) and the paired ones computed with SS, using the complete list of coefficients (62).


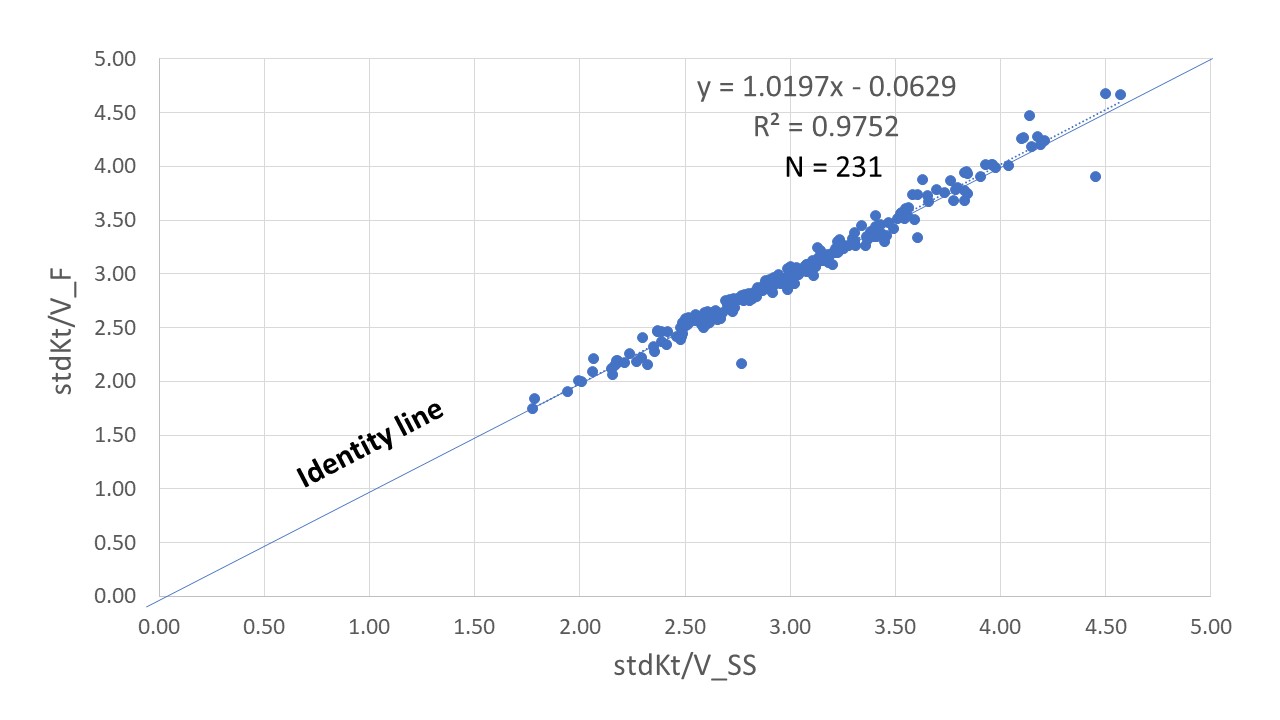


Figure 2S. Agreement plot (Bland-Altman) of stdKt/V values estimated with the formula (F) and the paired ones computed with SS, using the complete list of coefficients (62).


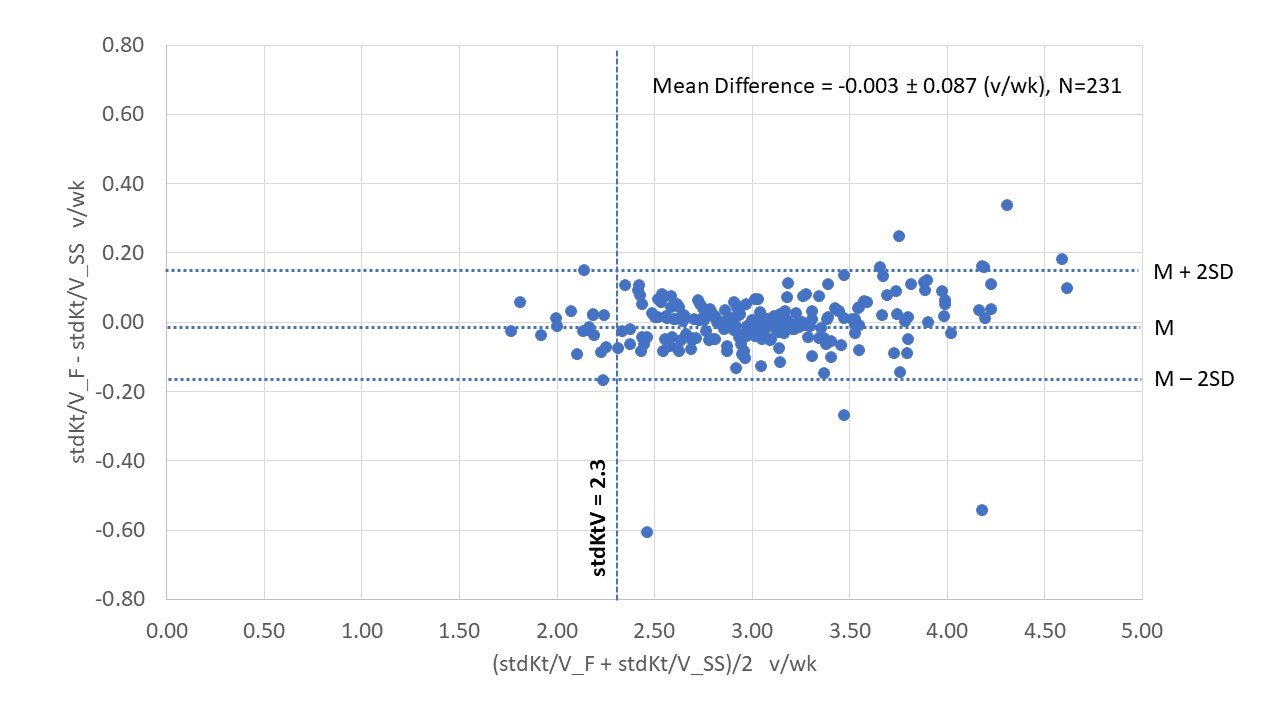


Explanatory Note to Table 4

The number **212** in the third row of Table 4 ("To be prescribed") is correct, as far as UKM is concerned. Figure 4 shows that any anuric patient receiving an eKt/V ≥ 0.6 will achieve a stdKt/V ≥ 2.3 v/wk. In this way, if an eKt/V of at least 0.6 is delivered, it is not necessary to prescribe 6 or 7 sessions per week to achieve the target stdKt/V.

We prepared the following input data set for the “What-if” software (10):

Frank50,1,12345,0,25,7,250,180,150,7,50,kd,110

Frank60,1,12345,0,30,7,250,180,150,7,60,kd,130

Frank70,1,12345,0,35,7,250,180,150,7,70,kd,152

Frank80,1,12345,0,40,7,250,180,160,7,80,kd,161

Frank90,1,12345,0,45,7,250,180,180,7,90,kd,161

We assumed: 5 sessions per week, Kru=0, eKt/V=0.6, and V=0.50 x post-dialysis body weight (varying from 50 to 90 kg)- As expected, despite variations in the patient body weight, to simulate the dialysis prescription in a variety of individuals with different V, the What-if software shows that an eKt/V 0f 0.6 in anuric patients provides a stdKt/V of 2.3 v/wk (10) (Figure 3S).


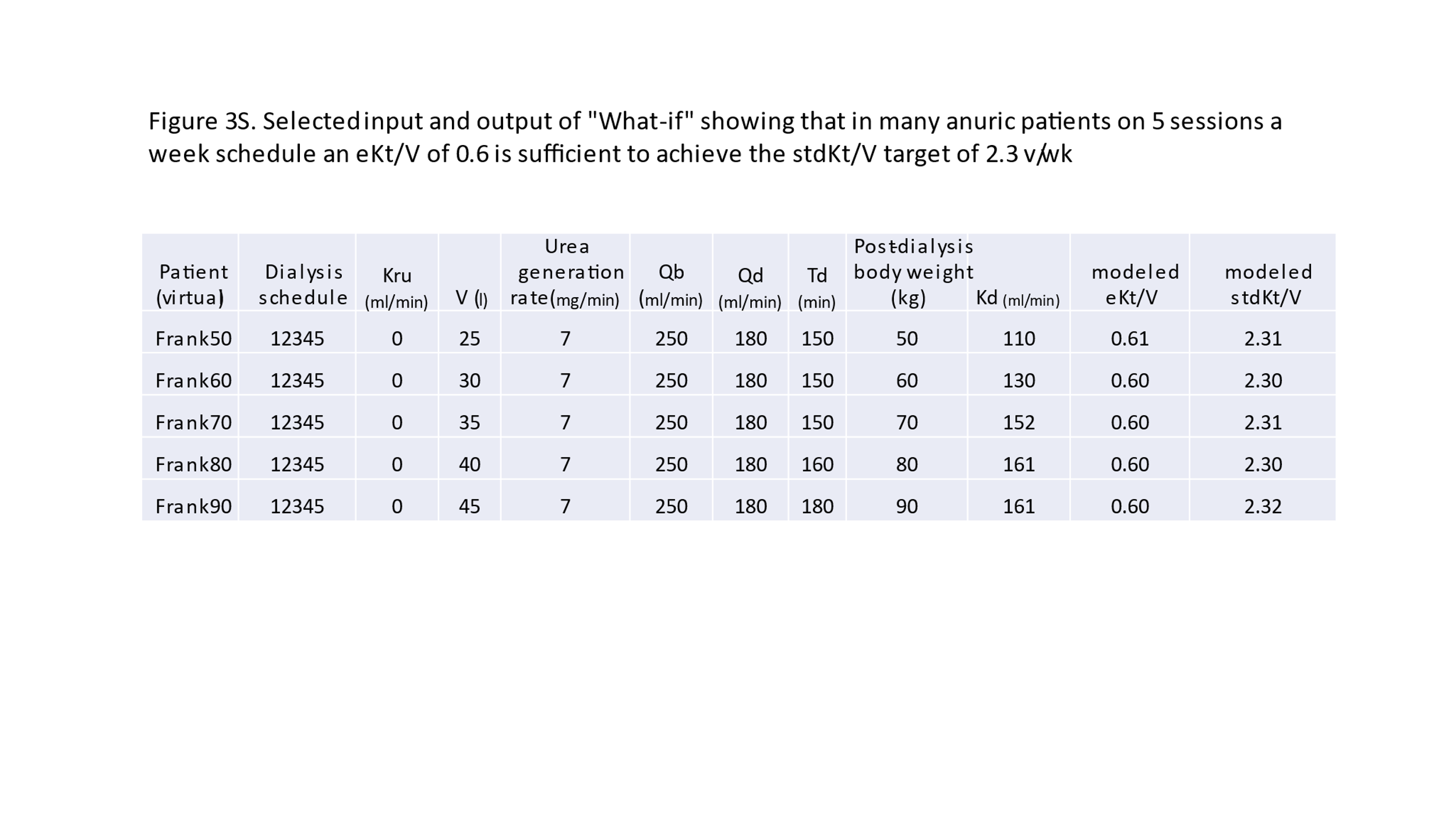

Supplement: gfad212_Supplemental_File [file gfad212_supplemental_file.docx]
